# Impact of age on degenerative joint disease of the temporomandibular joint: A systematic review and meta-analysis

**DOI:** 10.1097/MD.0000000000041915

**Published:** 2025-04-25

**Authors:** Zhiyuan Wu, Yi Lin, Youlai Lin

**Affiliations:** a Department of Stomatology, Fujian Provincial Hospital, Fuzhou, Fujian, China.

**Keywords:** age, degenerative joint disease of the temporomandibular joint, influencing factors, meta-analysis, temporomandibular joint

## Abstract

**Background:**

It is unclear that the influence of age on degenerative joint disease (DJD) of the temporomandibular joint (TMJ).

**Methods:**

Relevant literature was retrieved from PubMed, Elsevier, Web of Science, and Google Scholar. EndNote 21 was used to consolidate the literature retrieved from these databases. Key information were extracted from the included studies, statistical analysis was performed using Stata 15.0. The quality of the studies was evaluated using the cross-sectional study evaluation criteria recommended by the Agency for Healthcare Research and Quality.

**Results:**

A total of 11 studies involving 2832 participants (1099 males, 1744 females) were included. The incidence of DJD of the TMJ was approximately 35% among individuals aged 20 to 39, 43% among those aged 40 to 59, and 54% among those aged 60–69.

**Conclusion:**

Age progression is a key risk factor for the development of DJD of the TMJ. The incidence of DJD of the TMJ increases progressively across different age groups, with a significant rise observed in middle to older age groups.

## 1. Introduction

The TMJ is a vital structure that connects the mandible to the skull. It not only supports and stabilizes the mandible, but also plays a key role in various oral and maxillofacial functions,such as chewing, swallowing, speech, and facial expression.^[1,2]^ TMJ-DJD is an umbrella term for degenerative changes in the structure and function of the TMJ,^[3]^ primarily encompassing osteoarthrosis and osteoarthritis. Osteoarthrosis refers to non-inflammatory lesions characterized by the wear and degeneration of joint tissues, accompanied by the formation of new bone on the joint surface. Clinically, it manifests as disc displacement or perforation, joint noise, and limited mandibular movement, but without pain in the joint area and masticatory muscles.^[4,5]^ Osteoarthritis, a type of low-inflammatory arthritis, involves the softening and wear of articular cartilage, thinning of cartilage, destruction of subchondral bone, and marginal bone growth, often accompanied by synovial inflammation. Its clinical features resemble osteoarthrosis but are distinguished by pain in the joint and masticatory muscle areas, as well as loss of joint function.^[6,7]^ As a significant public health issue, TMJ-DJD affects approximately 5% to 12% of the general population and is the leading cause of non-dental-related chronic pain in the oral and facial region.^[8]^ With aging, the TMJ may undergo degenerative changes, leading to a series of clinical symptoms such as joint pain, clicking, and limited mouth opening, which can severely impact patients’ quality of life.^[9]^ In recent years, as the population ages, the incidence of TMJ-DJD has gradually increased, making it a key focus in the field of oral medicine.^[10]^

Although numerous studies on TMJ-DJD have been conducted, many focus on specific age group or particular symptom, lacking a comprehensive analysis across different age groups. Therefore, this study aims to comprehensively assess the impact of age on TMJ-DJD through a systematic review and meta-analysis, quantifying the relationship between age and TMJ-DJD, and clarifying its incidence, clinical manifestations, and prognostic characteristics across different age groups.

## 2. Materials and methods

### 2.1. Literature search

A systematic literature search was conducted in PubMed, Elsevier, Web of Science, and Google Scholar (see supplement materials, Supplemental Digital Content, https://links.lww.com/MD/O718). The following keywords were used: “Temporomandibular Joint,” “Age Factor,” and “ Degenerative Joint Disease,”Boolean operators (AND, OR, NOT) and advanced search filters available on the databases’ official websites were used to further narrow the search scope, limiting results to specific study types, journals, and publication dates. Additional articles were identified from reference lists during full-text review. The search was completed by October 2024.

### 2.2. Inclusion and exclusion criteria

The inclusion criteria for the selected studies were as follows: primary research, including clinical trials, cohort studies, cross-sectional studies, case-control studies; studies that explicitly involve patients with TMJ-DJD, including TMJ osteoarthritis, TMJ disorder syndrome, degenerative osteoarthropathy; studies that included patients across a wide age range; availability of complete data, including patient patient demographics (such as age, gender), disease characteristics (severity and specific type of TMJ degeneration), and research results (incidence rate, bone changes).

Studies were excluded if they met any of the following conditions: duplicate publications or similar content; incomplete, unclear, or obviously incorrect data; non-primary research; focusing solely on specific age groups (e.g., children, adolescents, or the elderly); lowe quality evaluations or insufficient methodological rigor.

### 2.3. Literature screening and data extraction

The variables for data extraction were clearly defined, including age, gender, and specific indicators of TMJ-DJD. A standardized data extraction form was developed, and 2 independent researchers performed the data extraction.

For literature screening, EndNote 21 was used to merge literature retrieved from different databases, and duplicate literature was removed using the “Find Duplicates” function. The collected literature was then reviewed to assess eligibility, and the full text of eligible studies was obtained for further analysis. Two researchers independently reviewed and screened the collected literature. Any discrepancies in either the data extraction or eligibility screening process were resolved through discussion. If agreement could not be reached, a third, more experienced researcher was consulted.

### 2.4. Literature quality evaluation

The quality of the selected studies was assessed using the cross-sectional study evaluation criteria recommended by the Agency for Healthcare Research and Quality (AHRQ). These criteria consist of 11 items, each evaluated as “yes,” “no,” or “unclear.” a score of 1 is assigned for “yes,” while both “no” and “unclear” received a score of 0. the total possible score is 11, with studies categorized as follows: 0 to 3 indicates low quality, 4 to 7 medium quality, and 8 to 11 high quality.

### 2.5. Statistical analysis

Review Manager 5.3 software was used for the combined analysis. For dichotomous variables, odds ratios (OR) were calculated, while standardized mean differences were used as the effect size for continuous variables. Heterogeneity among the included studies was assessed using the I² value and other relevant indicators. In cases of significant heterogeneity, subgroup analysis was conducted to investigate potential sources of variation. If heterogeneity could not be resolved, a random-effects model was used to calculate the combined effect size.

## 3. Results

### 3.1. Inclusion of literature

**Figure 1.**
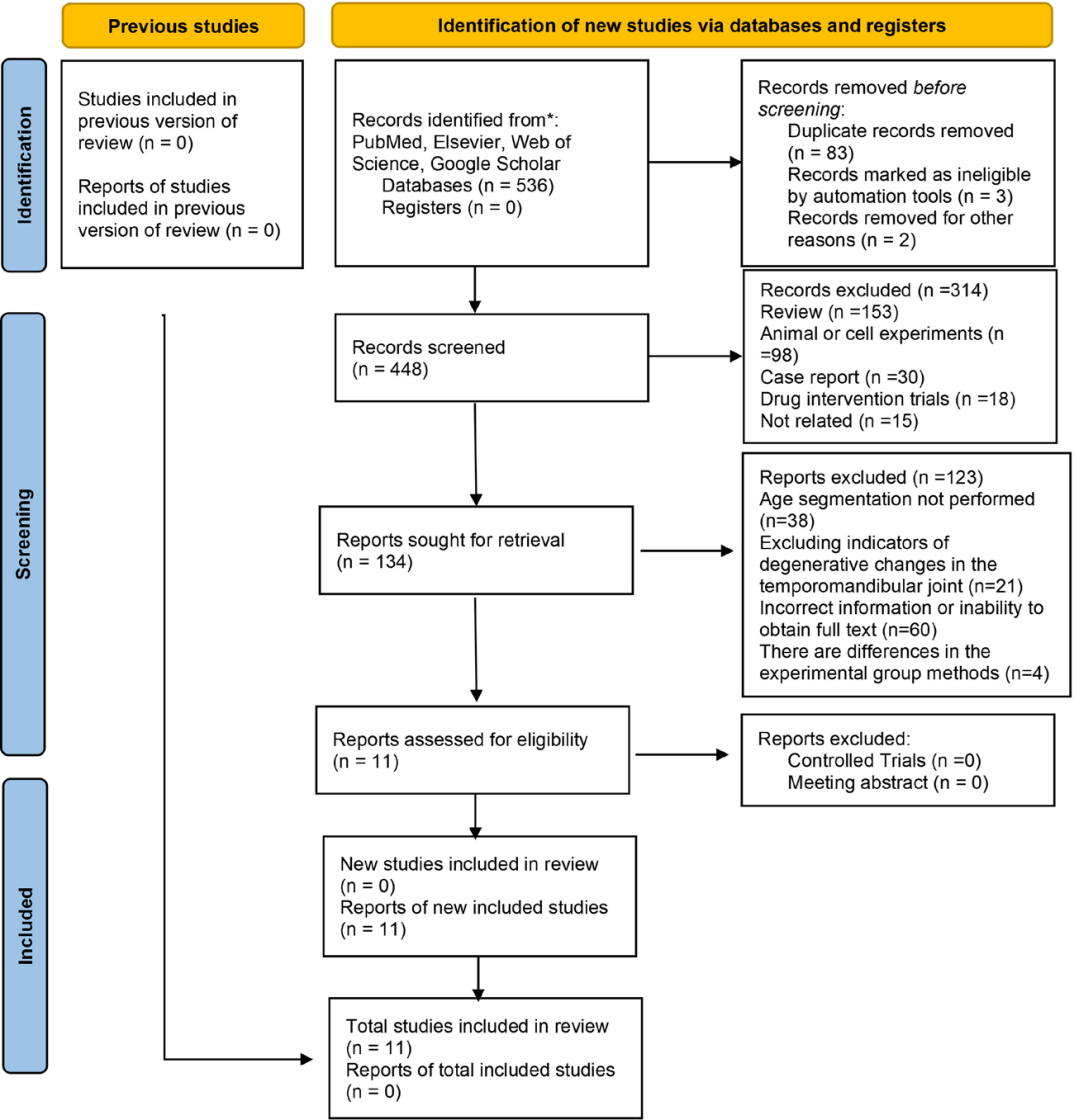
Literature inclusion process.

A total of 538 studies were identified through database searches. After removing 83 duplicate records, 455 studies remained for further evaluation. These were subsequently screened, resulting in the exclusion of 314 studies that were deemed irrelevant based on title and abstract review. The full text of the remaining 134 articles was assessed, and studies that did not meet the predefined outcome criteria or lacked age stratification were excluded. Ultimately, 1 studies were included in the final analysis. The detailed selection.process is shown in Figure 1.

A total of 11 studies^[11–21]^ were included, comprising 2832 research subjects, of whom1,099 were males and 1744 were females. The primary TMJ–DJD conditions examined were osteoarthritis and osteoarthropathy, with cone beam computed tomography (CBCT) being the predominant diagnostic method. The populations included in the selected literature originated from diverse countries, including Sweden, the United States, Thailand, Turkey, Brazil, and South Korea. Most samples were collected from living individuals, with only 2 studies^[17,21]^ using postmortem samples. Detailed study characteristics are presented in Table 1.

**Table 1:** Basic characteristics of the included studies.

Author and year	Sample source	Specific disease types	Inspection method	Number of sample	Male	Female	Crowd	Research type
Alexiou 2009^[11]^	Living	Osteoarthritis	CBCT	71	11	60	Sweden	Cross-sectional study
Alzahrani 2020^[12]^	Living	Osteoarthritis	CBCT	145	54	91	USA	Retrospective cross-sectional study
Arayasantiparb 2020^[13]^	Living	Osteoarthritis and osteoarthritis	CBCT	73	6	67	Thailand	Cross-sectional study
Borahan 2016^[14]^	Living	Osteoarthritis	CBCT	795	369	426	Türkiye	Cross-sectional study
dos Anjos Pontual 2012^[15]^	Living	Degenerative bone changes	Magnetic Resonance	319	69	250	Brazil	Cross-sectional study
Gorurgoz 2023^[16]^	Living	Degenerative bone changes	CBCT	258	110	148	Türkiye	Cross-sectional study
Ishibashi 1995^[17]^	Corpse	Degenerative bone changes	Magnetic Resonance	34	33	12	-	Anatomical research
Koc 2020^[18]^	Living	Osteoarthritis	CBCT	150	43	107	Türkiye	Cross-sectional study
Simonek 2024^[19]^	Living	Degenerative bone changes	Panoramic X-ray film	600	317	283	Switzerland	Cross-sectional study
Song 2024^[20]^	Living	Osteoarthritis	CBCT	183	39	144	the republic of korea	Cross-sectional study
Wiese 2008^[21]^	Corpse	Temporomandibular joint disorder	CBCT	204	48	156	Sweden	Anatomical research

CBCT = cone beam computed tomography.

### 3.2. Assessment of literature

The quality of the included studies was assessed using the cross-sectional study evaluation criteria recommended by AHRQ. All studies scored above 8 points, demonstrating high-quality standards.. Therefore, the overall quality of the literature included in this article is relatively high, as shown in Table 2.

**Table 2:** Quality assessment of included studies using AHRQ criteria.

Author and year	Q1	Q2	Q3	Q4	Q5	Q6	Q7	Q8	Q9	Q10	Q11	Total score
Alexiou 2009^[11]^	1	1	0	1	1	0	1	1	1	1	1	9
Alzahrani 2020^[12]^	1	1	0	1	1	1	1	1	1	1	0	9
Arayasantiparb 2020^[13]^	1	1	1	1	0	0	1	1	1	1	0	8
Borahan 2016^[14]^	1	1	1	1	1	1	1	1	1	1	1	11
dos Anjos Pontual 2012^[15]^	1	1	0	1	1	1	1	1	1	1	1	10
Gorurgoz 2023^[16]^	1	1	1	1	1	1	1	1	1	1	0	10
Ishibashi 1995^[17]^	1	1	1	1	1	1	1	1	1	1	1	11
Koc 2020^[18]^	1	1	1	1	1	1	1	1	1	1	1	11
Simonek 2024^[19]^	1	1	1	1	1	1	1	1	1	1	1	11
Song 2024^[20]^	1	1	1	1	0	1	1	1	1	1	1	10
Wiese 2008^[21]^	1	1	1	1	1	1	1	1	1	1	1	11

AHRQ = the Agency for Healthcare Research and Quality.

### 3.3. Meta-analysis results

A total of 11 articles^[11–21]^ were included to explore the impact of age on TMJ–DJD. Among them, 6 studies^[11,13,14,18,19,21]^ provided detailed data on the incidence of TMJ–DJD across different age groups. The age groups were categorized as 20 to 29, 30 to 39, 40 to 49, 50 to 59, 60 to 69, and 70 to 79 years old. I^2^ > 90%, with high heterogeneity. The incidence of tmj-djd in all age groups was combined by using the random effect model. The combined findings from these 6 studies indicate that the incidence of TMJ-DJD was approximately 35% between 20 and 39 years old, around 43% aged between 40 and 59 years old, and approximately 54% between 60 and 69 years old. A significant upward trend in the incidence of TMJ-DJD with increasing age was observed. The results showed that the heterogeneity of the MRI subgroup was reduced to 0%, indicating that the imaging examination of the mandibular joint is likely to be the source of heterogeneity (see Table 3 for details).

**Table 3:** Combined results of DJD of the temporomandibular joint incidence across different age groups.

Age group	Subgroup	Effect (95% Cl)	*I * ^2^	*P*
20 to 29	CBCT	0.23 (0.10–0.36)	91.4%	<.001
	MRI	0.59 (0.47–0.71)	0.0%	.812
	Total	0.347 (0.196–0.498)	93.3%	<.001
30 to 39	CBCT	0.35 (0.13–0.56)	96.4%	<.001
	MRI	0.68 (0.59–0.77)	0.0%	.485
	Total	0.345 (0.146–0.545)	97.3%	<.001
40 to 49	CBCT	0.31 (0.15–0.46)	91.5%	<.001
	MRI	0.73 (0.62–0.83)	0.0%	.388
	Total	0.424 (0.227–0.621)	95.4%	<.001
50 to 59	CBCT	0.31 (0.15–0.46)	91.6%	<.001
	MRI	0.74 (0.62–0.85)	0.0%	.336
	Total	0.427 (0.238–0.616)	95.0%	<.001
60 to 69	CBCT	0.44 (0.14–0.74)	97.6%	<.001
	MRI	0.76 (0.64–0.88)	0.0%	.938
	Total	0.537 (0.292–0.783)	96.7%	<.001
70 to 79	CBCT	0.37 (0.18–0.56)	92.1%	<.001
	MRI	0.79 (0.66–0.92)	0.0%	.768
	Total	0.46 (0.283–0.708)	94.3%	<.001

CBCT = cone beam computed tomography, CI = confidence interval, DJD = degenerative joint disease, MRI = magnetic resonance imaging.

**Figure 2.**
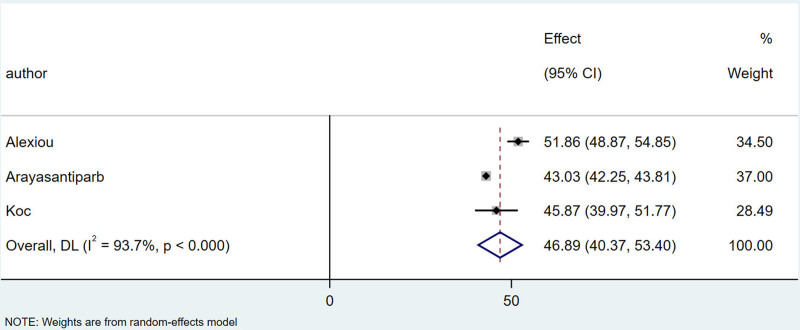
Forest plot of combined mean age for patients with temporomandibular joint DJD. DJD = degenerative joint disease.

**Figure 3.**
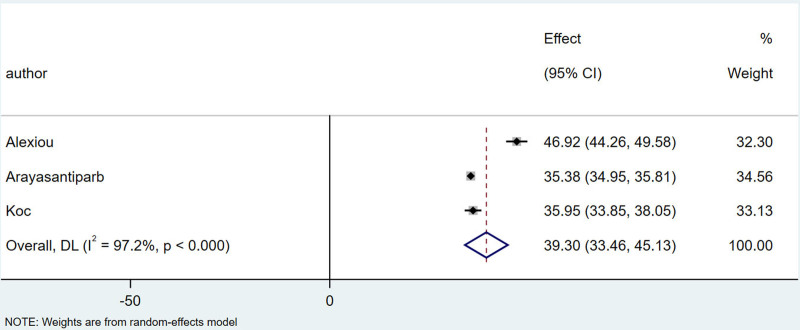
Forest plot of combined mean age for patients without temporomandibular joint DJD. DJD = degenerative joint disease.

Among the 11 articles, 3 studies^[15,16,20]^ provided comparisons of the mean age and standard deviation between patients with and without TMJ–DJD. I^2^ = 93.7%， There is a high degree of heterogeneity, which is combined by the random effect model, the combined results showed that the mean age of patients with TMJ-DJD was 46.89 years, with a 95% confidence interval (CI) of (43.37, 53.40). In contrast, the mean age of patients without TMJ-DJD was 39.30 years, with a 95% CI of (33.46, 45.13), which was significantly lower than that of the TMJ-DJD group. The specific details are illustrated in Figures 2 and 3.

**Figure 4.**
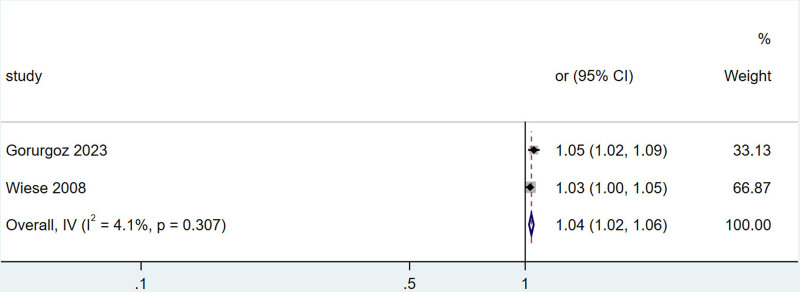
Forest plot of the meta-analysis on the association between age and temporomandibular joint DJD. DJD = degenerative joint disease.

Additionally, 2 studies^[16,21]^ provided the odds ratio (OR) for the effect of age on the occurrence of TMJ–DJD. There was no significant heterogeneity, and the fixed effect model was used for consolidation. The combined analysis yielded an OR of 1.04, with a 95% CI of (1.02, 1.06) and a *P*-value of 0.307. This indicates that age is a risk factor for TMJ-DJD, with an increasing incidence as age rises. The specific details are illustrated in Figure 4.

## 4. Discussion

The TMJ is primarily composed of the mandibular condyle and the glenoid fossa of the temporal bone, along with the articular disc located between them.^[22]^ The joint capsule, which is relatively loose, attaches to the bony surface surrounding the glenoid fossa and the periphery of the articular disc.^[23]^ The articular disc is anatomically divided into the anterior band, intermediate zone, posterior band, and bipartite zone, serving key functions such as shock absorption, stabilization of the joint, and adjustment of joint movement.^[24]^ The TMJ plays a crucial role in facilitating mandibular movements, including opening, closing, protrusion, and lateral movements, achieved through the coordinated sliding and rotation of the mandibular condyle within the glenoid fossa.^[25]^ Furthermore, the TMJ provides both stability and cushioning, thereby protecting the mandibular and temporal bones from injury.^[26]^

Temporomandibular joint-degenerative joint disease is a common oral disease with a complex etiology and a wide range of clinical manifestations. It encompasses various musculoskeletal and neuromuscular disorders affecting the masticatory muscles, TMJ, and related structures.^[27]^ CBCT is currently considered the most precise 3-dimensional imaging method for examining TMJ disorders,^[28]^ and magnetic resonance imaging (MRI) also demonstrates high sensitivity and specificity in TMJ-DJD patients, showing strong consistency with CBCT findings.^[29]^ Systematic reviews have indicated that the primary pathological changes in TMJ-DJD include condylar surface erosion, flattening, osteophyte formation, and sclerosis. In terms of anatomical features, smaller condylar size and a posteriorly positioned condylar head within the TMJ are commonly observed.^[30]^ Additionally, studies have suggested that the inclination of the articular condyle is associated with the morphology of the glenoid fossa and condylar fossa.^[5]^

Observation of these pathological changes is crucial for early diagnosis of TMJ-DJD, yet definitive diagnosis relies heavily on imaging findings.^[31]^ Therefore, identifying clinical symptoms to quickly identify high-risk populations and implement precise prevention measures is of great significance. Research has shown that TMJ clicking and pain are potential risk factors for TMJ-DJD, particularly in younger individuals.^[32]^ Although there is some evidence linking TMJ-DJD with pain, a clear causal relationship has not been firmly established.^[33]^

When examining the impact of age on TMJ-DJD, it is generally accepted among scholars that increasing age is associated with a higher incidence of TMJ-DJD. However, some studies have suggested that age is not related to TMJ-DJD. For example, Crusoe-Rebello et al^[34]^ examined 144 joints and found no association between age and the incidence of bony changes. Similarly, Isberg et al^[35]^ found no significant difference in the risk of articular disc displacement between males and females, and no difference in the risk of disc displacement or pain intensity between adolescents and the elderly. Therefore, there is no consensus has been reached regarding the relationship between the characteristics of TMJ-DJD and age across different age groups.

Understanding the clinical manifestations, imaging features of tmj-djd and its relationship with age factors has important clinical relevance for guiding clinical treatment, evaluating prognosis and formulating preventive measures. Through in-depth study of the pathogenesis and clinical characteristics of TMJ-DJD, we can provide patients with more accurate and effective treatment, so as to improve their quality of life.

Gorurgoz^[16]^ and Wiese^[21]^ indicated that there is a linear relationship between age and the incidence of TMJ-DJD, with a higher incidence observed in older individuals. Furthermore, Gorurgoz^[16]^ noted that the relationship between age and the incidence of right TMJ-DJD is stronger than that of the left TMJ, suggesting an asymmetry between the 2 joints. The combined results of 3 studies^[11,13,18]^ showed that the average age of patients with TMJ-DJD was 46.89 years, significantly higher than the average age of 39.30 years for patients without TMJ-DJD. While these studies highlight a strong association between TMJ-DJD and age, they do not compare incidence rates across different age groups.

The combined analysis of 6 studies in this paper revealed that the incidence of TMJ-DJD is approximately 35% among individuals aged 20 to 39, around 43% among those aged 40 to 59, and approximately 54% among those aged 60 to 69. These findings suggest that the relationship between age and the TMJ-DJD incidence is not strictly linear. Specifically, the incidence is around 35% for both the 20 to 29 and 30 to 39 age groups, and around 43% for both the 40 to 49 and 50 to 59 age groups, indicating a stepwise increase in the incidence with age.

Although the incidence of TMJ-DJD is relatively low among younger populations, a notable proportion of individuals are still affected, which may be related to changes in modern lifestyles, poor chewing habits, and work stress.^[36]^ Therefore, TMJ health should be taken seriously among younger populations, with a focus on prevention and education. As individuals enter the 40 to 59 age range, the incidence of TMJ-DJD significantly rises to approximately 43%, likely due to age- related physiological changes such as the progressive degeneration of articular cartilage, decreased ligament elasticity, and laxity of the joint capsule with advancing age.^[37]^ Moreover, individuals in this age group may face additional oral health risks, such as periodontal disease and tooth loss, which could adversely affect TMJ health.^[38]^

In the 60 to 69 age group, the incidence of TMJ-DJD further increases to 54%, indicating that degenerative changes in the TMJ become widespread at this stage. widespread at this stage, older adults in this age range may face an increased risk of joint-related conditions, such as osteoarthritis and joint capsule fibrosis, which can impair joint function and diminish quality of life.^[39]^ However, in the 70 to 79 age group, the incidence of TMJ-DJD decreases to 46%, with no further increase. The observed decrease in TMJ-DJD incidence among individuals aged 70 to 79 may reflect survivorship bias, as healthier individuals with intact joint function are more likely to survive into advanced age. Additionally, reduced diagnostic sensitivity in older populations (e.g., underreporting due to comorbidities) or adaptive pain tolerance mechanisms might contribute to this trend.^[40]^ Therefore, the development of personalized prevention strategies, the enhancement of public education, and improvements of early diagnosis and treatment across different age groups are of great significance for maintaining TMJ health and improving the quality of life for the entire population.

In addition to age, the incidence of TMJ-DJD is closely linked to gender differences and asymmetries between the left and right mandibular joints. These complex factors further complicate the pathogenesis and preventive strategies of this disease. Regarding gender differences, most studies consistently indicate that women are more susceptible to degenerative changes in the TMJ compared to men.^[41–43]^ This gender predisposition may result from the interplay of multiple physiological and psychological factors. Women generally have weaker masseter muscle strength and greater joint flexibility, which may potentially affect mandibular stability and increase the risk of degenerative changes.^[44]^

Furthermore, women often face higher psychological stress, including anxiety and depression, which have been shown to exacerbate TMJ disorder symptoms such as pain and dysfunction.^[45]^ Hormonal fluctuations, such as those occurring during pregnancy and menopause, may also adversely affect the metabolism of joint tissues, thereby promoting degenerative changes in the TMJ.^[46]^

With respect to differences between the left and right mandibular joints, although research findings are varied, some studies have suggest that degenerative changes are more commonly observed in the left mandibular joint.^[47]^ This left-right asymmetry may be related to factors such as individual chewing preferences, malocclusion, and even habitual posture.^[48]^ Long-term unilateral chewing, may lead to increased mechanical stress on the ipsilateral TMJ, accelerating articular cartilage wear and degeneration.^[49]^

These findings advocate for age-stratified screening protocols, particularly for individuals over 40, using CBCT or MRI to detect early degenerative changes. Clinicians should prioritize patient education on TMJ health maintenance (e.g., avoiding parafunctional habits) and consider age as a criterion for initiating prophylactic interventions, such as occlusal splints or physical therapy, in high-risk populations. For older adults (≥60 years), multidisciplinary management addressing concurrent conditions (e.g., osteoporosis, periodontal disease) may mitigate TMJ-DJD progression.

While chronological age is a robust proxy for cumulative joint stress, biological aging processes (e.g., hormonal decline, oxidative stress) and lifestyle factors (e.g., diet, occupational jaw loading) likely interact with age to drive TMJ-DJD. Future studies should incorporate biomarkers of aging (e.g., telomere length, inflammatory cytokines) and longitudinal designs to disentangle these effects. Additionally, the lack of data on comorbidities (e.g., diabetes, rheumatoid arthritis) limits causal inference.

## 5. Conclusion

In summary, this paper provides a systematic review and meta-analysis of existing literature to examine the impact of age on TMJ-DJD. The findings indicate a significant correlation between age and the incidence of TMJ-DJD; however, the relationship is not strictly linear. Across different age groups, the incidence of TMJ-DJD increases in a stepwise manner, with a marked increase in middle and old age groups. This finding emphasizes that as individuals age, they should pay more attention to the health of their TMJ and highlights the importance of personalized prevention and treatment strategies for different age groups.

Nonetheless, this study also has certain limitations. While age is a key factor influencing TMJ-DJD, others factors such as gender, genetic background, lifestyle, and chewing habits, may also play a significant role in the development of degenerative changes. Since this study primarily focused on age, it did not comprehensively explore the interactions between these factors. Therefore, future research is expected to incorporate a broader range of studies to explore the specific mechanisms by which multiple factors contribute to TMJ-DJD, with the goal in of developing more precise and effective prevention and treatment strategies.

## Author contributions

**Conceptualization:** Youlai Lin, Zhiyuan Wu, Yi Lin.

**Data curation:** Youlai Lin, Zhiyuan Wu, Yi Lin.

**Formal analysis:** Zhiyuan Wu, Yi Lin.

**Funding acquisition:** Youlai Lin.

**Investigation:** Youlai Lin, Zhiyuan Wu, Yi Lin.

**Methodology:** Youlai Lin, Zhiyuan Wu, Yi Lin.

**Software:** Zhiyuan Wu.

**Supervision:** Youlai Lin, Zhiyuan Wu.

**Writing – review & editing:** Zhiyuan Wu, Yi Lin.

**Writing – original draft:** Youlai Lin, Zhiyuan Wu, Yi Lin.

## Supplementary Material


