# Supplementary material for: Impact of age on degenerative joint disease of the temporomandibular joint: A systematic review and meta-analysis
Source: Medicine (Baltimore). 2025 Apr 25;104(17):e41915. doi: 10.1097/MD.0000000000041915 (PMC12040012; doi:10.1097/MD.0000000000041915)
Supplement: Supplementary file 1 [file medi-104-e41915-s001.docx]

| **Supplement materials: Search strategy.** | | | |
| --- | --- | --- | --- |
| **Search platform** | **Search term** | **Search method** | **Time frame** |
| PubMed | "Temporomandibular Joint," AND "Age Factor," AND " Degenerative Joint Disease," | All fields | Literature search until October 2024 |
| Elsevier | "Temporomandibular Joint," AND "Age Factor," AND " Degenerative Joint Disease," | One box search | Literature search until October 2024 |
| Web of science | "Temporomandibular Joint," AND "Age Factor," AND " Degenerative Joint Disease," | Any field | Literature search until October 2024 |
| Google Scholar | "Temporomandibular Joint," AND "Age Factor," AND " Degenerative Joint Disease," | One box search | Literature search until October 2024 |
